# Efficacy of Short-Term High-Dose Statin in Preventing Contrast-Induced Nephropathy: A Meta-Analysis of Seven Randomized Controlled Trials

**DOI:** 10.1371/journal.pone.0034450

**Published:** 2012-04-12

**Authors:** Yongchuan Li, Yawei Liu, Lili Fu, Changlin Mei, Bing Dai

**Affiliations:** Division of Nephrology, Nephrology Institute of PLA, Shanghai Changzheng Hospital, Second Military Medical University, Shanghai, China; The University of Manchester, United Kingdom

## Abstract

**Background:**

A few studies focused on statin therapy as specific prophylactic measures of contrast-induced nephropathy have been published with conflicting results. In this meta-analysis of randomized controlled trials, we aimed to assess the effectiveness of shor-term high-dose statin treatment for the prevention of CIN and clinical outcomes and re-evaluate of the potential benefits of statin therapy.

**Methods:**

We searched PubMed, OVID, EMBASE, Web of science and the Cochrane Central Register of Controlled Trials databases for randomized controlled trials comparing short-term high-dose statin treatment versus low-dose statin treatment or placebo for preventing CIN. Our outcome measures were the risk of CIN within 2–5 days after contrast administration and need for dialysis.

**Results:**

Seven randomized controlled trials with a total of 1,399 patients were identified and analyzed. The overall results based on fixed-effect model showed that the use of short-term high-dose statin treatment was associated with a significant reduction in risk of CIN (RR = 0.51, 95% CI 0.34–0.76, p = 0.001; I^2^ = 0%). The incidence of acute renal failure requiring dialysis was not significant different after the use of statin (RR = 0.33, 95% CI 0.05–2.10, p = 0.24; I^2^ = 0%). The use of statin was not associated with a significant decrease in the plasma C-reactive protein level (SMD −0.64, 95% CI: −1.57 to 0.29, P = 0.18, I^2^ = 97%).

**Conclusions:**

Although this meta-analysis supports the use of statin to reduce the incidence of CIN, it must be considered in the context of variable patient demographics. Only a limited recommendation can be made in favour of the use of statin based on current data. Considering the limitations of included studies, a large, well designed trial that incorporates the evaluation of clinically relevant outcomes in participants with different underlying risks of CIN is required to more adequately assess the role for statin in CIN prevention.

## Introduction

Contrast-induced nephropathy (CIN), characterized by the development of acute renal failure after exposure to radiocontrast, is the third leading cause of hospital-acquired acute renal injury, accounting for 11% of all cases [1]. It is defined as an increase in baseline serum creatinine level of 25% or an absolute increase of 44 µmol/L (0.5 mg/dL). Although CIN is generally benign in most instances, it is associated with lengthened hospital stays, increased health care costs, and higher risk of death [2]–[4]. Several strategies, including using iso-osmolar contrast, limiting the amount of administered contrast media and volume expansion have become well established methods for the prevention of CIN.

The pathophysiological mechanisms of CIN is not well known. However, multiple studies have suggested that renal vasoconstriction, oxidative stress, inflammation and direct tubular cell damage by contrast media may play crucial important roles in the renal injury process [5]–[8]. Statins, drugs primarily associated with low-density lipoprotein cholesterol-lowering effects, have been shown to possess pleiotropic effects that include enhancement of endothelial nitric oxide production [9]–[11], anti-inflammatory and antioxidative actions [12], [13]. Therefore, statins are considered as promising candidate agents for the prevention of CIN.

A few studies focused on statin therapy as specific prophylactic measures of CIN have been published with conflicting results [14]–[22]. In this meta-analysis of randomized controlled trials (RCTs), we aimed to assess the effectiveness of short-term high-dose statin treatment for the prevention of CIN and clinical outcomes and re-evaluate of the potential benefits of statin therapy.

## Materials and Methods

### Search strategy

The literature search was performed on PubMed (1966- October 2011), OVID (1966 to October 2011), EMBASE (1966- October 2011), Web of science (1986- October 2011) and the Cochrane Central Register of Controlled Trials (1996 to October 2011). We derived three comprehensive search themes that were then combined using the Boolean operator “AND”. For the theme “contrast media”, we used combinations of MeSH, entry terms and text words: contrast, radiocontrast, contrast medium, contrast media, contrast dye, radiographic contrast, radiocontrast media, radiocontrast medium and contrast agent. For the theme “renal insuficiency”, we used: renal insufficiency, renal failure, diabetic nephropathies, nephritis, nephropathy, nephrotoxic, (impair or injury or damage or reduce) and (renal or kidney), contrast-induced nephropathy and contrast-associated nephropathy. For the theme “statin”, statin, atorvastatin, rosuvastatin, cerivastatin, simvastatin, pravastatin, lovastatin, Hydroxymethylglutaryl(HMG)-CoA reductase inhibitors and HMG-CoA reductase inhibitors were used. Appendix S1 shows the detailed search method. We did not restrict by language or type of article. To identify other relevant studies, we manually scanned reference lists from identified trials and review articles, and we also searched conference proceedings. We requested original data by directly contacting authors.

### Study selection

We included studies when the following criteria were met: (1) randomized, controlled trials assessing preventive strategies for CIN; (2) the intervention was high-dose statin (defined as a daily dose of 80 mg or 40 mg) versus low-dose statin treatment (defined as a daily dose of 20 mg or 10 mg) or placebo. Studies that incorporated NAC were included only if both arms were administered NAC; (3) studies reported the incidence of contrast-induced nephropathy in both arms. We did not restrict eligibility according to kidney function. The primary outcome measure was the development of contrast-induced nephropathy, defined as an increase in baseline serum creatinine level of 25% or an absolute increase of 44 µmol/L (0.5 mg/dL) within 2 to 5 days after the exposure to contrast medium. Secondary outcome measures were need for dislysis, in-hospital mortality and length of hospital stay.

### Data extraction and quality assessment

Data were collected independently by 2 reviewers. Extracted data included patient characteristics (mean age, diabetes status, mean baseline creatinine level and postprocedural change in C-reactive protein level); inclusion criteria; type and dose of contrast media; protocol for the treatment of statins; periprocedural hydration protocol and specific definition of CIN. Quality assessment was judged on concealment of treatment allocation; similarity of both groups at baseline regarding prognostic factors; eligibility criteria; blinding of outcome assessors, care providers, and patients; completeness of follow-up; and intention-to-treat analysis [23]. We quantified study quality by using the Jadad score [24]. A third reviewer adjudicated any disagreement about extracted data. Then data were checked and entered into the Review Manager (Version 5.0. Copenhagen: The Nordic Cochrane Centre, The Cochrane Collaboration, 2008) database for further analysis.

### Statistical analysis

Dichotomous data (contrast-induced nephropathy and need for dialysis) were analyzed using the risk ratio (RR) measure and its 95% confidence interval (CI). Moreover, heterogeneity across trials was evaluated with I^2^ statistic, which defined as I^2^>50%. If heterogeneity existed, a random-effect model was used to assess the overall estimate. Otherwise, a fixed-effect model was chosen. We assessed for potential publication bias by using Begg funnel plots of the natural log of the relative risk versus its standard error [25]. To further detect and evaluate clinically significant heterogeneity, we also a priori decided to perform several subgroup analyses to identify potential differences in treatment across the trials. Subgroup analysis was conducted based on renal function in participants at baseline (with or without renal impairment), the control group property (low dose of statin or control), the addition of NAC (with or without NAC), and Jadad study quality score (Jadad>3 or Jadad≤3). All tests were two-tailed and a P value less than 0.05 was regarded as significant in this meta-analysis.

## Results

### Selected studies and characteristics

**Figure 1:**
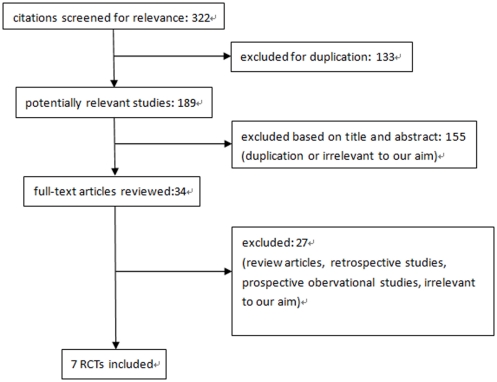
Study selection diagram.

We identified 322 potentially relevant citations from the initial literature search. After independently reviewing the title and abstract of all potential articles, 34 articles were considered of interest and reviewed in full-text. Of these, 27 were excluded from the meta-analysis (review articles, retrospective studies, prospective obervational studies, irrelevant to our aim). Although the study carried out by Acikel Sadik et al [20] did not provide data on the incidence of CIN, we requested it by directly contacting the author. Therefore, seven randomized controlled studies with a total of 1,399 patients with undergoing radiocontrast-related procedures were identified and analyzed [16]–[22]. Our search strategy is outlined in Figure 1.

Table 1 and table 2 summarizes the characteristics of the included studies. All of them had been reported since 2008. 693 subjects were assigned to short-term high-dose statin treatment group and 706 subjects were assigned to short-term low-dose or non-statin treatment group. The proportion of patients lost to follow-up was less than 5% in all studies. CIN was defined differently among the included studies. Six studies [16], [17], [19]–[22] used an increase in serum creatinine of >0.5 mg/dL or >25% from baseline within 48–72 h after radiocontrast exposure as their definition, whereas the other study [18] regarded an absolute increase in serum creatinine of >0.5 mg/dl within 5 days as their primary definition of CIN. Two studies [17], [18] involved patients with creatinine clearance rate less than 60 ml/min; four studies [16], [20]–[22] enrolled patients with creatinine clearance rate or estimated glomerular filtration rate>60 ml/min and there was no restriction according to renal function but patients with creatinine level >3 mg/dl were excluded in the study by Patti G et al [19]. All studies evaluated patients undergoing coronary angiography or other intervention, for example, percutaneous coronary intervention (PCI). All of the patients received low-osmolar or iso-osmolar contrast media and median contrast volume ranged from 93 ml to 240 ml. Periprocedural hydration was used in every one, except the patients without pre-existing renal failure in the study by Patti G et al [19]. Five studies [16], [18]–[20], [22] used atorvastatin and simvastatin was used in the other two studies [17], [21]. The duration of statin treatment ranged from 3 to >7 days and the total dose ranged from 140 mg to >460 mg in the high-dose statin treatment group. Two of the included studies [16], [18] also used oral N-acetylcysteine (600 mg or 1200 mg) twice daily in both arms, started the day before the procedure. Allocation concealment and blinding were used in three studies [17]–[19] and the quality characteristics of the studies were shown in table 3.

**Table 1:** Characteristics of included studies.

Author, year	Patients,n		Inclusion criteria	Statin protocol	Control	Contrast type	Median contrast volume,ml		Hydration procedure
	Statin	Control					Statin	Control	
Sang-Ho Jo et al,2008	118	118	CAG.SCr≥1.1 mg/dL or CrCl≤60 mL/min	Simvastatin,40 mg every 12 hours, 1 day pre-procedure and 1 day post-procedure	Placebo	Iodixanol	173	191	Isotonic saline,1 mg/kg/hour for 12 h before and 12 h after procedure
Anna Toso et al,2009	152	152	CAG and/or PCI.CrCl<60 ml/min	Atorvastatin,80 mg/day 2 days pre-procedure and 2 days post-procedure+NAC,1200 mg bid from 1 day before to 1 day post-procedure	Placebo+NAC, 1200 mg bid from 1 day before to 1 day post-procedure	Iodixanol	151	164	NS,1 ml/kg/hour for 12 h before and after the procedure
Xinwei et al,2009	113	115	PCI	Simvastatin, 80 mg/day from admission to the day before, 20 mg/day after procedure	Simvastatin, 20 mg/dayfrom admission to the end	Iodixanol for CKD,iohexol for others	227	240	NS, 1 ml/kg/hour for 6 to 12 hours before and 12 hours after procedure
Zhou Xia et al,2009	50	50	CAG or PCI	Atorvastatin,80 mg/day before for 1day,10 mg/day for 6days after procedure	Atorvastatin, 10 mg/day for 7 days	Iopamidol	119	113	1000 mL saline infusion, for 12 hours before and 12 hours after intervention
Sadik Acikel et al,2010	80	80	CAG.eGFR>60 ml/min per 1.73 m^2^	Atorvastatin,40 mg/day,3 days pre-procedure and 2 days post-procedure	Nothing	Iohexol	105	103	Isotonic saline,1 ml/kg/hour starting 4 h before and continuing until 24 h after procedure
Hakan Ozhan et al,2010	60	70	CAG.SCr≤1.5 mg/dl or eGFR≥70 ml/min per 1.73 m^2^	Atorvastatin,80 mg 1 day pre-procedure and 2 days post-procedure+600 mg NAC bid pre-procedure	600 mg NAC bid pre-procedure	Iopamidol	97	93	1000 ml saline infusion during 6 h after procedure
Giuseppe Patti et al,2011	120	121	CAG and/or PCI.SCr≤3 mg/dl	Atorvastatin,80 mg(12 hs before)+40 mg(2 hs before), 40 mg for 2days after procedure	Placebe+40 mg atorvastatin for 2days after procedure	Iobitridol	209	213	For patients CrCl<60 ml/min,1 ml/hour/kg for 12 h before and 24 h after intervention

Statin = statin-treated group(high-dose);Control = control group(low-dose or non-statin);CAG = coronary angiography;PCI = percutaneous coronary intervention;CrCl = creatinine clearance;Scr = serum creatinine;eGFR = estimated glomerular filtration rate;NAC = N-acetylcysteine;NS = 0.9% sodium chloride.

**Table 2:** Characteristics of included studies-continued.

Author, year	Mean age,y		Diabetic patients,%		Mean baseline sCr level,µmol/L (mg/dL)		Postprocedural changes in CRP levels, mg/L (Mean±SD)		Definition of CIN	Events,n	
	Statin	Control	Statin	Control	Statin	Control	Statin	Control		Statin	Control
Sang-Ho Jo et al,2008	65	66	28.2%	23.6%	114(1.286)	110(1.248)	1.25±1.25	1.27±1.79	Increase of Scr>0.5 mg/dL or >25% within 48 hours	3	4
Anna Toso et al,2009	75	76	20%	22%	106(1.2)	104(1.18)	NS	NS	Increase of Scr≥0.5 mg/dl within 5 days.	15	16
Xinwei et al,2009	65	66	20%	22%	72(0.82)	73(0.83)	1.9±0.5	3.4±1.2	Increase of Scr>0.5 mg/dL or >25% within 48 hours	6	18
Zhou Xia et al,2009	60	61	22%	18%	92(1.04)	95(1.08)	NS	NS	Increase of Scr>0.5 mg/dL or >25% within 72 hours	0	3
Sadik Acikel et al,2010	59	61	23.8%	25.0%	74(0.84)	75(0.85)	NS	NS	Increase of Scr>0.5 mg/dL within 48 hours	0	1
Hakan Ozhan et al,2010	54	55	15.00%	17.14%	77.8(0.88)	77.8(0.88)	NS	NS	Increase of Scr>0.5 mg/dL or >25% within 48 hours	2	7
Giuseppe Patti et al,2011	65	66	30%	25%	92(1.04)	92(1.04)	8.4±10.5	13.1±20.8	Increase of Scr>0.5 mg/dL or >25% within 48 hours	6	16

Statin = statin-treated group (high-dose);Control = control group (low-dose or non-statin);CAG = coronary angiography;PCI = percutaneous coronary intervention;CrCl = creatinine clearance;Scr = serum creatinine;CRP = C-reactive protein;eGFR = estimated glomerular filtration rate;NAC = N-acetylcysteine;NS = 0.9% sodium chloride; NS = not specified or available.

**Table 3:** Quality of included RCTs.

Author, Year	Jadad Score	Allocation Concealment	Similarity of Baseline Characteristics	Eligibility Criteria	Blinding			Completeness of Follow-up	Intention-to- Treat Analysis
					Outcome Assessor	Care Provider	Patient		
Sang-Ho Jo et al,2008	5	YES	YES	YES	NS	YES	YES	YES	YES
Anna Toso et al,2009	5	YES	YES	YES	NS	YES	YES	YES	YES
Xinwei et al,2009	3	YES	YES	YES	NO	NO	NO	YES	NS
Zhou Xia et al,2009	3	NS	YES	YES	NS	NS	NS	YES	NS
Sadik Acikel et al,2010	1	NS	NO	YES	NO	NO	NO	YES	NS
Hakan Ozhan et al,2010	2	NS	YES	YES	NO	NO	NO	YES	NS
Giuseppe Patti et al,2011	5	YES	YES	YES	YES	YES	YES	YES	YES

**NS = not specified or available.**

### Effects of statin treatment on clinical outcomes

**Figure 2:**
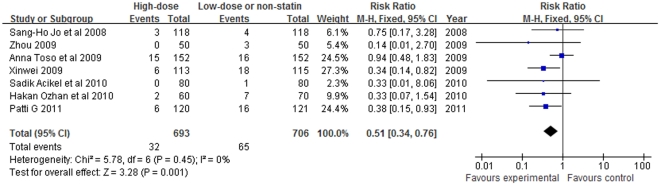
Forest plot of risk ratios and 95% confidence intervals (CI) for the incidence of contrast induced nephropathy among patients assigned to statin therapy versus control.

The overall results based on fixed-effect model showed that the use of short-term high-dose statin treatment was associated with a significant reduction in risk of CIN (RR = 0.51, 95% CI 0.34–0.76, p = 0.001; I^2^ = 0%; Figure 2). The incidence of acute renal failure requiring dialysis was very low and was not significant different after the use of statin (3 studies [17]–[19], RR = 0.33, 95% CI 0.05–2.10, p = 0.24; I^2^ = 0%).

In-hospital mortality was observed in only one patient who died from acute heart failure aggravated by major bleeding in these seven studies [18]. Although the study carried out by Zhou Xia et al [22] reported incidence of cardiovascular event in short-term high-dose treatment group (5/50) and low-dose group (2/50), it didn't give any details. The total length of hospital stay were reported only in two studies. There was no difference between statin-treated group and control group in length of hospital stay in the study [17] by Jo SH et al. However, length of stay after intervention was shorter in patients randomized to atorvastatin (2.9±0.9 vs 3.2±0.8 days, P = 0.007) in the other study [19].

**Figure 3:**
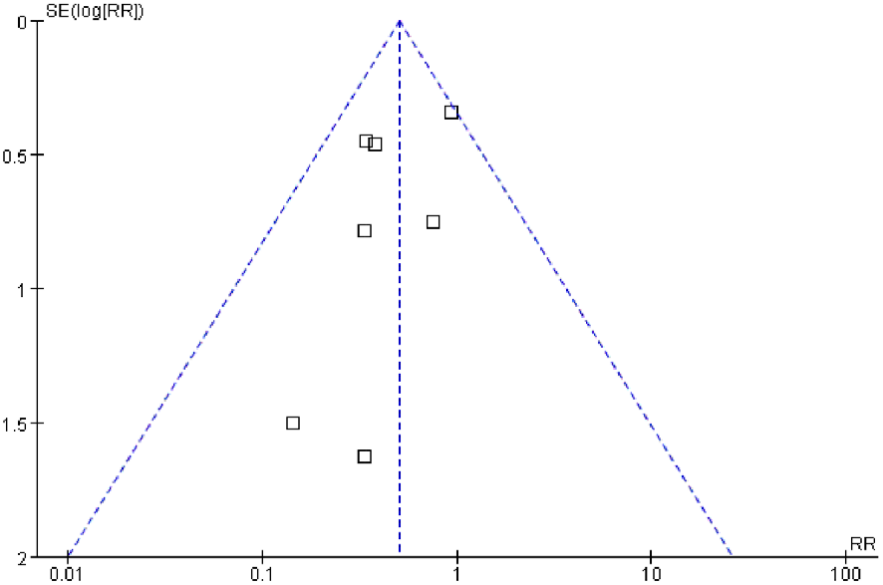
Funnel plot with 95% confidence intervals (CI) to assess for evidence of publication bias.

Figure 3 demonstrates that there was no evidence to suggest publication bias according to the relative symmetry in the Begg funnel plot.

Postprocedural changes in C-reactive protein (CRP) levels were analyzed in three trials [17], [19], [21]. The use of statin was not associated with a significant decrease in the plasma CRP level (SMD −0.64, 95% CI: −1.57 to 0.29, P  = 0.18, I^2^ = 97%).

### Subgroup analysis

**Figure 4:**
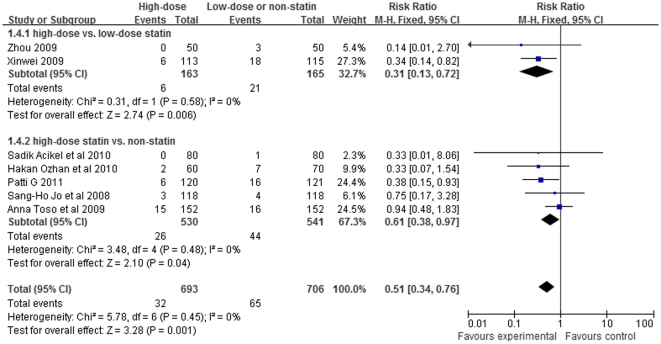
Forest plot of risk ratios and 95% confidence intervals (CI) for the incidence of CIN among patients assigned to short-term high-dose statin treatment versus low-dose or non-statin.

Classified according to low-dose statin-treated or not in control group, studies [16]–[20] comparing short-term high-dose statin treatment with non-statin treatment showed a significant protective trend toward decreased incidence of CIN (RR = 0.61, 95%CI 0.38–0.97, P = 0.04; Figure 4) and the same effect was seen in other two studies [21], [22] which compared short-term high-dose with low-dose statin treament (RR = 0.31, 95%CI 0.13–0.72, P = 0.006).

**Figure 5:**
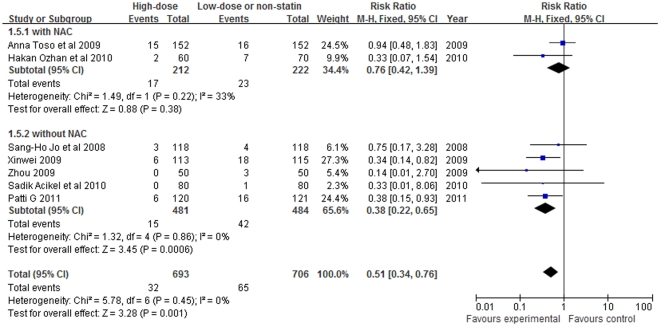
Forest plot of risk ratios and 95% confidence intervals (CI) for the incidence of CIN among patients assigned to statin therapy versus control with NAC using or not.

In all five studies in which statin was compared with control without the addition of NAC, the risk of CIN was significantly decreased (RR = 0.38, 95%CI 0.22–0.65, P = 0.0006; Figure 5). In contrast, the risk of CIN did not significantly differ in the two studies in which statin plus NAC versus NAC only (RR = 0.76, 95%CI 0.42–1.39, P = 0.38).

**Figure 6:**
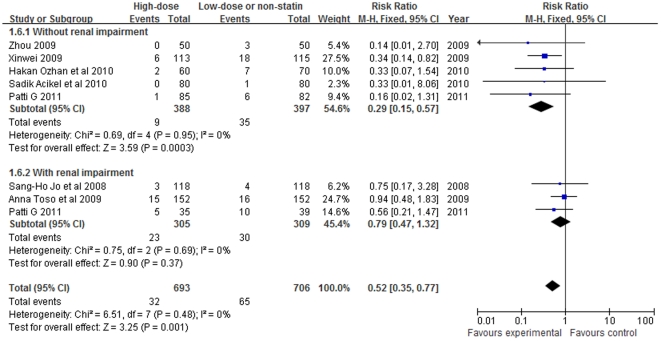
Forest plot of risk ratios and 95% confidence intervals (CI) for the incidence of CIN among patients assigned to statin therapy versus control according to renal function.

In studies that included patients without renal impairment at baseline (creatinine clearance rate or estimated glomerular filtration rate>60 ml/min), RR was 0.29 (95%CI 0.15–0.57, P = 0.0003; Figure 6). A reduced risk of CIN was not found in studies that included patients with pre-existing renal impairment (creatinine clearance rate ≤60 ml/min). RR for CIN associated with the use of statin was 0.79 (95%CI 0.47–1.32, P = 0.37).

**Figure 7:**
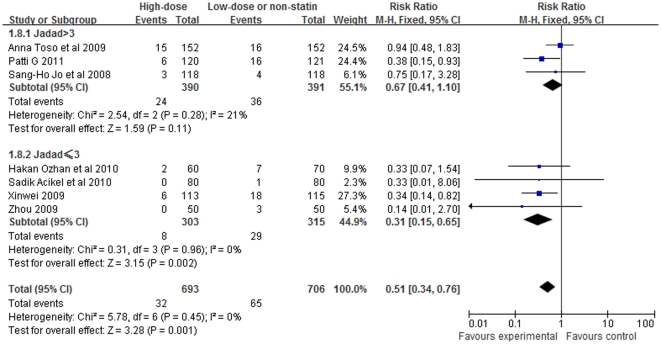
Forest plot of risk ratios and 95% confidence intervals (CI) for the incidence of CIN among patients assigned to statin therapy versus control according to Jadad score.

Classified according to the Jadad score >3 or not, studies whose Jadad score≤3 showed a significant reduction of CIN (RR = 0.31, 95%CI 0.15–0.65, P = 0.002; Figure 7). However, the risk of CIN did not significantly differ in the studies whose Jadad score>3 (RR = 0.67, 95%CI 0.41–1.10, P = 0.11).

## Discussion

In the past two decades, although hydration has been well recognized and widely performed to prevent the CIN, the incidence of CIN did not decrease. So the efficacy of many other interventions are still under testing. From 2004 to 2011, a few studies focused on using statin as a specific prophylactic measure of CIN prevention have been published. In this meta-analysis of 7 randomized controlled trials (RCTs), we found that statin could significantly reduce the risk of CIN without decreasing the incidence of death or need for dialysis. However, there was marked clinical heterogeneity among these studies, indicating the need for a large definitive RCT.

In addition to their intended impact on blood cholesterol levels, statins have been found to have multiple nonlipid-lowering effects, which include enhancement of endothelial nitric oxide production [9]–[11], anti-inflammatory and antioxidative actions [12], [13]. Given their pleiotropic effects, statins could decrease acute renal injury after iodinated contrast administration through two major pathways. Firstly, statins may modulate the kidney hypoperfusion after contrast administration by downregulation of angiotensin receptors and decreased synthesis of endothelin-1 [26], [27]. Secondly, toxic damage on the tubular cells by oxygen-free radicals and proinflammatory cytokines may be decreased by anti-inflammatory effects of statins that inhibit tissue factor expression by macrophages and prevent the activation of nuclear factor-κB [28]. Moreover, its nonlipid-lowering effect could be demonstrated within a few hours after statin therapy initiation [29], [30]. Although many clinical trials [31], [32] have shown that high-dose statins provide more clinical benefits, such as atorvastatin 80 mg can further reduce vascular risks compared with low-dose statin therapy, the threshold of statins to reduce the risks of CIN remains unknown. In this meta-analysis, all of the included trials were short-term high-dose statin therapy, two of which compared two different doses of statin in preventing CIN. We found that high-dose statin therapy significantly lowered the incident of CIN compared with low-dose statin therapy. These results were consistent with the previous studies that high-dose statin has been shown to be more potent to suppress platelet activity and inflammatory chemokines than low-dose statin therapy [33] ^.^

The results of this meta-analysis are not in line with research from Zhang T et al [34], Zhang L et al [35] and Pappy R et al [36] which showed non-statistically significant reduction in the incidence of CIN with statin treatment from the pooled estimate for the randomized trials. In fact, Zhang T et al [34] and Pappy R et al [36] included both randomized and non-randomized trials in their meta-analysis, while the latter might lead to potential bias because it was impossible to completely remove interference of unknown confounding factors. The meta-analysis by Zhang L et al [35] involved only 4 RCTs, which included an abstract that overlapped with participants included in a separate study by the same author. Therefore, to avoid including any individual participant more than once, abstract by the same author was excluded in our meta-analysis [37]. Moreover, all of above three meta-analysis did not include two large scale studies [19], [20] published in recent days.

Although the main conclusion in our meta-analysis was similar to that in the recent meta-analysis [38], [39], these similar results shall be treated with cautious interpretation. First, in our meta-analysis, we found that statin was able to prevent CIN only in studies with lower quality, especially those which did not use of blinding, but not effective in high quality studies. This indicated that the results from the meta-analysis could not definite the effects of statins in preventing CIN. Second, pre-existing renal dysfunction was known to be an independent predictor of CIN that occured in up to 15% of patients with chronic kidney disease (CKD). However, subgroup analysis in risk group for CIN also weakened our findings. The studies that included patients with pre-existing renal dysfunction found no preventive effect of statins. Multiple nonreversible pathogenetic mechanisms involved in advanced renal failure may attenuate the response for statins, especially for their vasodilatation and anti-inflammatory effects. In addition, although a higher serum level was expected in CKD patients, local drug concentration still might be compromised due to renal scar and structural impairment. So the safety, pharmacokinetics and permeability of various statins in CKD patients should be well evaluated in future studies. Third, N-acetylcysteine, a thiol-containing antioxidant, was a promising agent to prevent contrast induced nephropathy because of its antioxidative and haemodynamic effects in the renal medulla and its general organ-protective effects described in several ischaemia-reperfusion models [40]. In the subgroup analysis of statin plus NAC versus NAC only, the difference were not significant. This could be attributed to that statin and NAC might decrease CIN occurrence through the similar pathways, such as scavenging oxygen free radicals produced after contrast exposure; therefore, the second agent could not exert addictive renal protection if NAC offered full protection available through antioxidants.

There are several potential limitations in this meta-analysis. Firstly, although all included studies reported the incidence of CIN, few trials designed to investigate the effect of statins on hard clinical outcomes such as acute renal failure requiring dialysis, length of hospital stay and in-hospital mortality. Secondly, we did not have access to patient-level data to determine whether the risk factors (eg, diabetes and age) could influence the effect of short-term high-dose statin treatment on the risk of contrast-induced nephropathy. Finally, studies included in this meta-analysis analyzed the efficacy of statin with different type of statins for varied periods of time. It is possible that dose, duration and type of statin may have differential effect in prevention of CIN. An accepted uniform statin protocol would be helpful in both the clinical and research arenas.

In conclusion, although this meta-analysis supports the use of statin to reduce the incidence of CIN, this result must be considered in the context of variable patient demographics. Only a limited recommendation can be made in favour of the use of statin based on current data. Considering the limitations of included studies, a large, well designed trial that incorporates the evaluation of clinically relevant outcomes in participants with different underlying risks of CIN is required to more adequately assess the role for statin in CIN prevention.

## Supporting Information

Appendix S1 **Detailed search method.**
(DOC)
