# Supplementary material for: Efficacy of Short-Term High-Dose Statin in Preventing Contrast-Induced Nephropathy: A Meta-Analysis of Seven Randomized Controlled Trials
Source: PLoS One. 2012 Apr 12;7(4):e34450. doi: 10.1371/journal.pone.0034450 (PMC3325242; doi:10.1371/journal.pone.0034450)
Supplement: Appendix S1 — Detailed search method. (DOC) [file pone.0034450.s001.doc]

**APPENDIX: SEARCH STRATEGY**

**PubMed**

1. “Contrast Media” [MeSH]
2. Contrast
3. Radiocontrast
4. Contrast medium
5. Contrast media
6. Contrast dye
7. Radiographic contrast
8. Radiocontrast media
9. Radiocontrast medium
10. Contrast agent
11. #1 or #2 or #3 or #4 or #5 or #6 or #7 or #8 or #9 or #10
12. “Renal Insufﬁciency” [MeSH]
13. “Diabetic Nephropathies” [MeSH]
14. “Nephritis” [MeSH]
15. Nephritis
16. Nephropathy
17. Nephrotoxic
18. (impair or injury or damage or reduce) and (renal or kidney)
19. Contrast-induced nephropathy
20. Contrast-associated nephropathy
21. #12 or #13 or #14 or #15 or #16 or #17 or #18 or #19 or #20
22. “Hydroxymethylglutaryl-coa reductase inhibitors” [MeSH]
23. Statin
24. Atorvastatin
25. Rosuvastatin
26. Cerivastatin
27. Simvastatin
28. Pravastatin
29. Lovastatin
30. Hydroxymethylglutaryl-CoA reductase inhibitors
31. HMG-CoA reductase inhibitors
32. #22 or #23 or #24 or #25 or #26 or #27 or #28 or #29 or #30 or #31
33. #11 and #21 and #32

**Ovid**

1. exp contrast media/
2. (contrast or contrast media or contrast medium or contrast dye or radiographic contrast).tw.
3. (radiocontrast media or radiocontrast medium).tw.
4. contrast agent$.tw.
5. or/#1-4
6. exp nephritis/
7. exp Renal Insufﬁciency/
8. exp diabetic nephropathies/
9. (nephritis or nephropath$ or nephrotoxic$).tw.
10. ((impair$ or injur$ or damag$ or reduc$) adj2 (renal or kidney)).tw.
11. or/#6-10
12. #5 and #11
13. (contrast-induced nephr$ or contrast-associated nephr$).tw.
14. #12 or #13
15. exp hydroxymethylglutaryl-coa reductase inhibitors/
16. (statin or Atorvastatin or Rosuvastatin or Cerivastatin or Simvastatin or Pravastatin or Lovastatin or Hydroxymethylglutaryl-CoA reductase inhibitors or HMG-CoA reductase inhibitors).tw.
17. #14 and #16

**EMBASE**

1. ‘contrast’/exp and media
2. ‘contrast’/exp and medium
3. ‘contrast’/exp and ‘dye’/exp
4. radiographic and ‘contrast’/exp
5. radiocontrast and media
6. radiocontrast and medium
7. ‘contrast’/exp and agent
8. #1 or #2 or #3 or #4 or #5 or #6 or #7
9. renal and insufﬁciency
10. ‘diabetic’/exp and nephropathies
11. ‘nephritis’/exp
12. ‘nephropathy’/exp
13. Nephrotoxic
14. (impair or injury or damage or reduce) and (renal or ‘kidney’/exp)
15. ‘contrast induced’ and ‘nephropathy’/exp
16. ‘contrast associated’ and ‘nephropathy’/exp
17. #9 or #10 or #11 or #12 or #13 or #14 or #15 or #16
18. #8 and #17
19. Statin
20. Atorvastatin
21. Rosuvastatin
22. Cerivastatin
23. Simvastatin
24. Pravastatin
25. Lovastatin
26. Hydroxymethylglutaryl-CoA and reductase and inhibitors
27. HMG-CoA and reductase and inhibitors
28. #19 or #20 or #21 or #22 or #23 or #24 or #25 or #26 or #27
29. #18 and #28

**Web of Science**

1. (contrast or radiocontrast or contrast media or contrast medium or contrast dye or radiographic contrast or radiocontrast media or radiocontrast medium or contrast agent)
2. (nephritis or nephropathy or nephrotoxic or contrast-induced nephropathy or contrast-associated nephropathy)
3. (impair or injury or damage or reduce) and (renal or kidney)
4. #2 or #3
5. (statin or atorvastatin or rosuvastatin or cerivastatin or simvastatin or pravastatin or lovastatin or hydroxymethylglutaryl-CoA reductase inhibitors or HMG-CoA reductase inhibitors)
6. #1 and #4 and #5

**Conchrane Central Register of Controlled Trials**

1. Contrast Media explode all trees (MeSH)
2. (contrast or radiocontrast or contrast media or contrast medium or contrast dye or radiographic contrast or radiocontrast media or radiocontrast medium or contrast agent)
3. Renal Insufﬁciency explode all trees (MeSH)
4. Diabetic Nephropathies explode all trees (MeSH)
5. Nephritis explode all trees (MeSH)
6. (nephritis or nephropathy or nephrotoxic or contrast-induced nephropathy or contrast-associated nephropathy)
7. (impair or injury or damage or reduce) and (renal or kidney)
8. Hydroxymethylglutaryl-coa reductase inhibitors explode all trees (MeSH)
9. (statin or atorvastatin or rosuvastatin or cerivastatin or simvastatin or pravastatin or lovastatin or hydroxymethylglutaryl-CoA reductase inhibitors or HMG-CoA reductase inhibitors)
10. (#1 or #2)
11. (#3 or #4 or #5 or #6 or #7)
12. (#8 or #9)
13. (#10 and #11 and #12)
